# Absence of Relationship between Mitochondrial DNA Haplogroups and Cisplatin-Induced Hearing Loss

**DOI:** 10.1155/2017/5185268

**Published:** 2017-07-03

**Authors:** D. Graterol, C. Mir, C. Garcia-Vaquero, I. Braña, A. N. Pardo, M. I. Rochera-Villach, A. Lyakhovich, M. E. LLeonart, J. Lorente

**Affiliations:** ^1^Otolaryngology Department, Hospital Vall d'Hebron, Passeig Vall d'Hebron 119-129, 08035 Barcelona, Spain; ^2^Biomedical Research in Cancer Stem Cells, Pathology Department, Hospital Vall d'Hebron, Passeig Vall d'Hebron 119-129, 08035 Barcelona, Spain; ^3^Oncology Department, Hospital Vall d'Hebron, Passeig Vall d'Hebron 119-129, 08035 Barcelona, Spain; ^4^Anaesthesiology and Resuscitation Department, Hospital Vall d'Hebron, Passeig Vall d'Hebron 119-129, 08035 Barcelona, Spain; ^5^Group of Mitochondrial Dysfunction and Diseases, International Clinical Research Center and St. Anne's University Hospital Brno, Brno, Czech Republic

## Abstract

**Background:**

Many drugs used for cancer chemotherapy produce reactive oxygen species, thus leading to various complications including nephrotoxicity, cardiotoxicity, and ototoxicity.

**Objective:**

We have provided a haplogroup analysis of a cohort of cancer patients treated with chemotherapy and compared factors associated with associated hearing loss.

**Study Design and Methods:**

This observational cohort study includes a pure-tone audiometry of the patients who underwent chemotherapeutic treatment. Medical history, presence of risk factors for hearing loss, toxic habits, and association with haplogroups have been determined.

**Results:**

40% of patients developed hearing loss after administration of cisplatin, which was bilateral and symmetrical and of high frequencies. The most frequent haplogroup was H with a slight overexpression of groups V and K and a low frequency of groups J and T. No association of the haplogroup types with the hearing loss has been found; however age was revealed as an important determining factor.

**Conclusions:**

Ototoxicity caused by cisplatin is manifested as bilateral, symmetrical, and predominantly high frequency hearing loss. Although we did not find a strong correlation of haplogroups with ototoxicity, our results revealed the existence of a risk group of elderly patients over 60, which are more susceptible to hearing loss induced by cisplatin, than young adults, regardless of preexisting hearing loss.

## 1. Introduction

Human mitochondrial DNA (mtDNA) haplogroups (subclades) are defined as genealogical groups of mitochondrial variants sharing a common maternal ancestor [1]. It has been suggested that genetic polymorphisms within the mitochondrial genome might lead to mitochondrial dysfunction (MDF), including impaired energy generation, low membrane potential, decreased ATP production, and accumulation of reactive oxygen species (ROS), all of which may contribute to clinical phenotype [2]. Therefore, common variations of mtDNA may potentially reveal medical conditions as they modify the risk of some diseases. Recently reported studies provide a correlation of mtDNA haplogroups with neurodegeneration and Alzheimer's disease, cardiomyopathy, Parkinson's disease, osteoarthritis, and diabetes [3–7]. Importantly, all these disorders are accompanied by increased ROS, one of the major MDF markers.

Although mitochondria are both the main source and the main target of ROS, environmental ROS may be equally deleterious also by contributing to mtDNA/nDNA damage in a vicious circle. In particular, ROS deriving from the action of DNA cross-linking agents, such as cisplatin, represent a great obstacle towards cancer chemotherapy due to their side-effects which include cardiotoxicity, nephrotoxicity, and ototoxicity [8–10]. Drug-mediated ototoxicity, specifically cochleotoxicity, is a concern for patients receiving medications for the treatment of serious illness [11]. Approximately 20 to 40% of patients receiving cisplatin developed ototoxicity which usually manifests as bilateral, symmetrical, irreversible, and high frequency hearing loss [12].

It is still unclear what might be the risk factor(s) enabling predicting the ototoxicity. The toxicity of cisplatin has been shown to be dependent on genetic variations related to individual mutations or polymorphisms of mitochondrial enzymes that play a pivotal role in hearing loss. Polymorphisms of the glutathione S-transferase (GST) gene family members have been linked to cisplatin-mediated individual ototoxicity [13]. Aminoglycosides have shown that ototoxicity is linked to the A1555G mutation, whose expression has been suggested to confine the phenotypic severity of several mtDNA haplogroups [14]. For that reason, we have performed a study of a group of cancer patients who had undergone chemotherapeutic treatment with cisplatin in the oncology service of Vall d'Hebron Hospital (Barcelona) for the last decade. We have examined the clinical history of the above patients and compared factors associated with cisplatin-associated hearing loss and corresponding toxic habits.

## 2. Methods and Design

### 2.1. Study Design

This is a descriptive, observational cohort study involving adult cancer patients (>18 y.o.) of Spanish Caucasian origin. A group of patients who were treated with a course of anticancer therapy between January 2009 and January 2015 in the oncology department at the Vall d'Hebron Hospital in Barcelona were included in the study. A total of 72 individuals who received cisplatin were selected for this study. Those who required combination chemotherapy or had already started the treatment or had an otological disease as well as those who required a concomitant antineoplastic treatment were excluded from the study.

### 2.2. Audiometry

A pure-tone audiometry was performed before treatment initiation and at the end of the 3d dose treatment. For the calculation of hearing loss, the following frequencies were used: 125, 250, 500, 1000, 2000, 4000, and 8000 Hz. The airway was taken as a reference point for calculating the hearing loss as long as there was no more than 5 dB gap between airway and bone conductivity. The average decibel values of these frequencies taken by airway of pre- and posttreatment visit were obtained. Subsequently, both averages are subtracted. Hearing loss greater than 10 dB was counted as significant hearing loss.

### 2.3. Haplogroup Determination

The samples were haplogrouped by PCR amplification of short mtDNA fragments, followed by restriction enzyme analysis obtained from buccal mucosa, as described elsewhere [15]. The first 30 patient's samples were analyzed in the mitochondrial pathology unit of the Research Institute of Vall d'Hebron Hospital. The rest of the samples were processed in the Department of Biological Anthropology at the Autonomous University of Barcelona. Samples were processed as qualitative variables.

## 3. Results

### 3.1. Association of Bilateral, Symmetrical, and High Frequencies' Hearing Loss with Cisplatin Anticancer Therapy

We have performed an observational cohort study among patients treated with cisplatin (Table 1). The sample group consisted of 72 patients aging between 27 and 82 years and with an average age of 63.3 years of both male (62%) and female (32%). The 63.8% of patients were located within the group of 60 and 82 years. Examination of medical history revealed arterial hypertension (AH, 29%), dyslipidemia (DLP, 26%), diabetes mellitus (DM, 18%), heart disease (5.6%), hepatobiliary disease (2.8%), lung disease (4%), and anemia (1.4%). Smoking (65%), coffee (61%), alcohol (23%), and tea (6%) consumption appeared among the most common toxic habits. 32% of individuals displayed some previous hearing loss, and 32% reported being exposed to noise. All patients were diagnosed with different neoplasia, including oral cavity-pharynx-larynx (45 individuals), bladder (12), testis (8), cavum (5), and cervix (2) (Table 2). 32 patients (44.4%) were claimed for regularly taking, at least, one type of drug. The most frequently consumed drugs were ACE inhibitors (16 patients), followed by oral antidiabetic (10 patients), simvastatin (8 patients), diuretics (5 patients), and calcium (2 patients).

**Figure 1:**
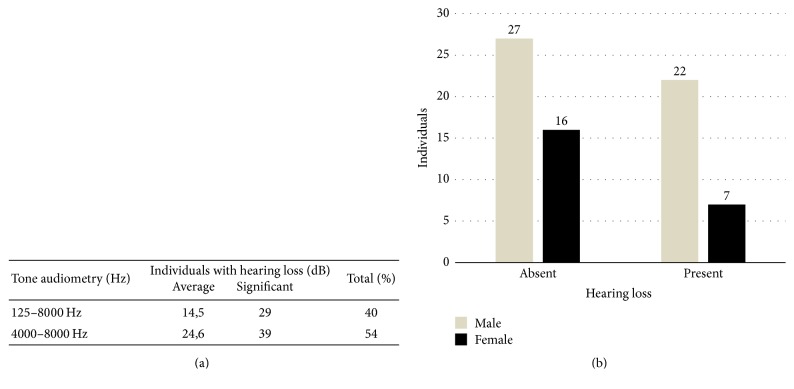
*Audiometric studies of cancer patients*. Hearing loss for cancer patients was analyzed by a pure-tone audiometry. The average decibel values were obtained for the following range of frequencies, 125–8000 Hz (125, 250, 500, 1000, 2000, 4000, and 8000), and were taken before and after treatment visit (a). The airway was taken as a reference for calculating the loss as long as there was no more than 5 dB gap between airway and bone conduction. Hearing loss was considered from a decrease in average greater than 10 dB and then was plotted onto the diagram as a male/female distribution (b).

Audiometric studies revealed that 40% of all patients who underwent chemotherapeutic treatment with cisplatin have developed hearing loss with an average loss of approximately 14.5 dB. The hearing loss was bilateral and symmetrical and of high frequencies. The percentage of individuals with hearing loss increased to 54% only if frequencies 4000 and 8000 Hz are taken into account. In this case, the average loss increases to 24.6 dB (Figure 1).

### 3.2. Haplogroup Analyses and Association with Hearing Loss

**Figure 2:**
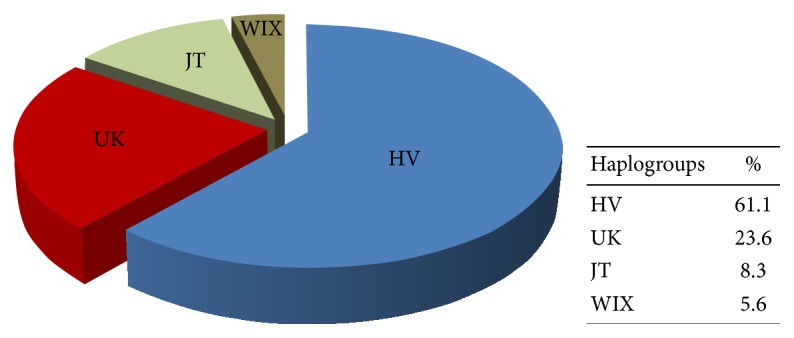
*Distribution of sample haplogroups among cancer patients*. A total of 72 individuals diagnosed with various cancers have been subjected to haplogroup analyses (%). JT includes haplogroups J, JT, and T. Haplogroup A is not included because of its low percentage.

In order to correlate cisplatin-mediated hearing loss with possible variations in mtDNA we performed analyses of haplogroups. Haplogroup H was the most common one among cancer patients (48.6%), followed by K (15.3%), V (12.5%), U (8.3%), WIX (5.6%), J (4.1%), JT (2.8%), A, and T groups with 1.4% (Figure 2). Although the sample size was not high enough to determine a statistically significant relationship between haplogroups and hearing loss (Table 3), we demonstrated that the elderly patients with a cutoff age over 60 were more susceptible to hearing loss induced by cisplatin than young adults, regardless of preexisting hearing loss.

## 4. Discussion

Prospective monitoring for ototoxicity allows for comparison of auditory outcomes across clinical trials, as well as for early detection, potential alterations in therapy, and auditory intervention and rehabilitation to ameliorate the adverse consequences of hearing loss [16]. Cisplatin-induced ototoxicity is being widely studied as one of the major side-effects in the course of the chemotherapeutic treatment of cancer patients. In our study, we have determined that 40% of patients had a significant hearing loss which was predominantly present at higher frequencies. We found no statistically significant association between hearing loss and haplogroups. However, we found a significant correlation of the hearing loss with age suggesting that adults or elder patients were more susceptible to cisplatin-induced hearing loss. These findings corroborate well with other studies [17, 18]. Importantly, other works not only indicate that older individuals are more prone to cisplatin-induced hearing loss but also demonstrate that children (<5 years) have more ototoxicity [19].

The most frequent haplogroup obtained in our study was H (48%), coinciding with the percentage of occurrence of haplogroups in Spain and Europe. This is not surprising as HV and branch haplogroups occur predominantly in Spain and Europe. However, we obtained a lower frequency for the haplogroups J and T as compared to Spanish and European media and an overexpression of haplogroups V and K. This difference seems to be attributed to a small sample size of already preselected (cancer) patients, as well as to a nonhomogeneous population that likely includes non-Spanish origin patients in the Barcelona area. Despite significant variability in the development of cisplatin ototoxicity, it is impossible to use it as a risk factor for cisplatin-induced hearing loss. However, it has been shown that the genetic characteristics of individuals, including point mutations of both nDNA and mtDNA, may affect response to certain drugs. Genetic predisposition to cisplatin-induced hearing loss can also be related to mitochondrial polymorphisms of the enzymes encoding antioxidative proteins. Furthermore, it has been observed that some patients with cisplatin ototoxicity are from the rare European mitochondrial haplogroup J, which has been associated with Leber's hereditary optic atrophy. Similarly, patients with testicular cancer undergoing cisplatin treatment showed differences in polymorphisms functional for glutathione S-transferase (GST) enzyme which is responsible for the phase II of drug metabolism.

Overall, provided a high enough sample size and proper controls, a study similar to ours may determine the predictors which should be taken into account when providing anticancer therapy. Eventually, combined screening for haplogroups and audiometric analyses may become part of the personalized medicine to define a risk group of patients for which a strategy other than chemotherapeutic treatment strategy should be applied.

## Figures and Tables

**Table 1:** Medical parameters of cancer patients undergoing cisplatin treatment^*∗*^.

Patients	Number	Age (average)	Systemic diseases (numbers)						An	Toxic Habits			HL	NE
			AH	DLP	DM	Hrt.	Lung	HB		Smoke	Coffee	Alc.		
Female	23	62.43	5	4	7	0	1	0	1	15	18	5	7	7
Male	49	63.79	16	15	6	4	2	2		32	26	12	16	16

Total	72	63,3	21 29%	19 26%	13 18%	4 6%	3 4%	2 2.8%		47 65%	44 61%	17 23%	23 32%	23 32%

^*∗*^AH, arterial hypertension; DLP, dyslipidemia; DM, diabetes mellitus; Hrt., heart; hepatobiliary (2.8%), lung (4%) diseases and anemia (1.4%). HL, previous hearing loss; NE, exposed to noise.

**Table 2:** Medical parameters of cancer patients including type of neoplasia and drug history^*∗∗*^.

Patients	Neoplasm spots					Drugs				
	OPL	Bld.	Tes.	CU	Cer.	ACE	OAD	Sim.	Diu.	Ca
Female	11	3		1	2	6	5	2	1	2
Male	34	9	8	4		10	5	6	4	0

Total	45	12	8	5	2	16	10	8	5	2

^*∗∗*^OPL, oral cavity, pharynx, and larynx; Bld., bladder; Tes., testis; CU, cavum; Cer. cervix; ACE, an angiotensin-converting-enzyme inhibitor; OAD, oral antidiabetic; Sim., simvastatin; Diu., diuretics; Ca, calcium.

**Table 3:** Distribution of hearing loss over haplogroups (%) (*p* = 0.796).

Haplogroup	Hearing loss		Total
	No	Yes	
H	65.71%	34.29%	48.61%
K	45.45%	54.55%	15.28%
U	66.67%	33.33%	8.33%
V	55.56%	44.44%	12.5%
Others	54.55%	45.45%	15.28%

Total	59.72%	40.28%	100%
